# A mixed methods study of Tai Chi exercise for patients with chronic heart failure aged 70 years and older

**DOI:** 10.1002/nop2.127

**Published:** 2018-02-21

**Authors:** Lena Hägglund, Kurt Boman, Margareta Brännström

**Affiliations:** ^1^ Department of Nursing Umeå University Umeå Sweden; ^2^ Department of Medicine‐Geriatric Skellefteå County Hospital Skellefteå Sweden; ^3^ Department of Public Health and Clinical Medicine Umeå University Umeå Sweden

**Keywords:** chronic heart failure, elderly, group training, mixed methods, Tai Chi

## Abstract

**Aims and objectives:**

This study aimed to evaluate Tai Chi group training among patients with chronic heart failure (CHF) aged 70 years and older.

**Background:**

Physical activity is recommended for CHF treatment. Tai Chi is found to be beneficial to different patient groups, although few studies focus on older patients with CHF.

**Design:**

A mixed methods study. Participants were randomly assigned to Tai Chi training twice a week for 16 weeks (*N *=* *25) or control (*N *=* *20). Quantitative data were collected at baseline, at the end of the training period and 6 months after training, assessing self‐rated fatigue and quality of life, natriuretic peptides and physical performance. Individual qualitative interviews were conducted with participants (*N *=* *10) in the Tai Chi training group.

**Results:**

No statistical differences between the Tai Chi training group and the control group in quality of life or natriuretic peptides was found. After 16 weeks, the training group tended to rate more reduced activity and the control group rated more mental fatigue. Participants in the training group rated increased general fatigue at follow‐up compared with baseline. Qualitative interviews showed that Tai Chi training was experienced as a new, feasible and meaningful activity. The importance of the leader and the group was emphasized. Improvements in balance were mentioned and there was no physical discomfort.

**Conclusion:**

Tai Chi was experienced as a feasible and meaningful form of physical exercise for patients with CHF aged over 70 years despite lack of achieved health improvement. Further investigations, using feasibility and meaningfulness as outcome variables seems to be useful.

## 1 INTRODUCTION

Chronic heart failure (CHF) is a common syndrome among older people, with a median prevalence rate of 11.8% for people 60 years or older (van Riet et al., 2016). Fatigue is one of the typical symptoms of CHF (Ponikowski et al., 2016) and by patients with CHF rated as one of the most distressing symptoms (Bekelman et al., 2011; Franzén, Blomqvist, & Saveman, 2006; Lam & Smeltzer, 2013; Zambroski, Moser, Bhat, & Ziegler, 2005). Exercise training has been shown to have beneficial effects among patients with CHF on self‐reported fatigue and dyspnoea (Pozehl, Duncan, & Hertzog, 2008). Tai Chi (Tai Chi Chuan) is a Chinese traditional mind‐body exercise that has met with increasing interest in Western countries. It is a combination of physical activity and muscle relaxation through slow, graceful movements which focus on breath control and mental concentration. Tai Chi can be understood as an aerobic exercise with meditative effects (Lan, Lai, & Chen, 2002).

## 2 BACKGROUND

Patients with CHF have reported their fatigue at similar levels as patients undergoing treatment for cancer (Fink, Sullivan, Zerwic, & Piano, 2009). High levels of fatigue is associated with a lower quality of life, worse perceived health and lower satisfaction with life among older adults with stable CHF (Stephen, 2008). In a qualitative study, the experience of fatigue is interpreted as an inside experience where the body is like a barometer for limitations in daily activities and an existential awareness of vulnerability and mortality (Jones, McDermott, Nowels, Matlock, & Bekelman, 2012).

In the 2016 European Society of Cardiology guidelines regular physical activity and structured exercise training is a Class l recommended treatment for patients with stable CHF (Ponikowski et al., 2016). However, there is still a lack of evidence concerning exercise among older patients with CHF since the mean age in most studies are younger than 70 years (Taylor et al., 2014). However, it is suggested that physical exercise can have similar benefits for older patients with CHF as for younger patients (Fleg, 2007). A training programme combining endurance exercise and resistance training had positive effects on physical capacity in a group of CHF patients aged 76 years (Pihl, Cider, Strömberg, Fridlund, & Mårtensson, 2011).

Furthermore, recommendations for physical exercise among older patients with CHF are poorly implemented in clinical practice. An important prerequisite to all therapeutic regimens is patient adherence and the problems with non‐adherence for physical exercise are well known (Conraads et al., 2012; Corotto, McCarey, Adams, Khazanie, & Whellan, 2013). Possible factors that can influence physical activity among patients with CHF are health aspects like experiences of adverse symptoms, such as breathlessness and fatigue and co‐morbidity, all which increase with age. Mental factors such as motivation and self‐image and social and environmental circumstances are also important (Tierney et al., 2011). In a review over strategies used to promote exercise adherence in people with HF, Tierney et al. (2012) underpinned the importance of using strategies that address motivation and experience of self‐efficacy. The authors also consider the use of alternative modes of activity for the CHF population.

In the last decade, several studies of various effects of Tai Chi exercise have been published. Systematic reviews have concluded that there is convincing evidence for the positive effects of Tai Chi on fall prevention (Huang, Feng, Li, & Lv, 2017), depressive symptoms (Chi, Jordan‐Marsh, Guo, Xie, & Bai, 2013) and cognitive performance (Wayne et al., 2014) among older people. Tendencies to improve physical performance by Tai Chi exercise was found in chronic conditions such as heart failure and chronic obstructive pulmonary disease (Chen, Hunt, Campbell, Peill, & Reid, 2015).

Tai Chi has even been found to be a safe form of physical exercise for patients with CHF, without reported side effects (Barrow, Bedford, Ives, O′Toole, & Channer, 2007; Yeh et al., 2004). Therefore, it can be recommended for this patient group (Cheng, 2007). A recent meta‐analysis of thirteen randomized controlled trials, including patients with CHF showed that participating in Tai Chi training significantly improved 6‐min walking distance and was beneficial to quality of life, left ventricular ejection fraction and N‐terminal pro‐brain natriuretic peptide (NTproBNP) (Gu et al., 2017). The mean age of participants in that meta‐analysis ranged from 51 to 76 years and only in one of the thirteen studies participants in the intervention group had a mean age over 70 years. The mean age of patients in primary health care with CHF is estimated to be around 79 years (Mosterd & Hoes, 2007; Olofsson, Edebro, & Boman, 2007) and to our knowledge no previous study of Tai Chi has focused on the oldest in this population, with age 70 years and older as inclusion criteria. Thus, there is a need to get more knowledge about the effects of exercise for groups of patients seldom included in clinical trials, such as older people (Piepoli et al., 2011). Our hypothesis was that the degree of self‐rated fatigue would be reduced, and health‐related quality of life would increase among the participants in the training group, compared with the control group.

The overall aim of this study was to evaluate Tai Chi group training among patients with CHF aged 70 years and older. This study aimed to:

- Explore fatigue, quality of life, physical performance and NTproBNP between groups of patients with CHF aged 70 years and older randomly assigned to Tai Chi training or a control group.
- Describe participants’ experiences of Tai Chi group training.

## 3 DESIGN, PROCEDURE AND PARTICIPANTS

A mixed methods study was conducted. As participation in a training programme is supposed to be a complex phenomenon, a combination of quantitative and qualitative data collection was used. Findings from both a group perspective and individual experiences are supposed to ensure a broader understanding of the study question (Halcomb & Hickman, 2015).

Inclusion criteria were verified chronic heart failure (LVEF <50%) in accordance with ESC guidelines for diagnosis (Ponikowski et al., 2016), stable medication, perceived fatigue and age 70 years or older. Exclusion criteria were unstable angina pectoris, myocardial infarction within the last 3 months, cognitive impairment or no perceived fatigue.

Participants were recruited from patient registers at three hospitals in three different cities in northern Sweden. One hundred and ninety‐one patients, 127 men and 64 women, were invited to the study by letters followed by a telephone call. Thirteen patients were not available by telephone. Forty‐five patients, 35 men and 10 women, agreed to participate.

**Figure 1:**
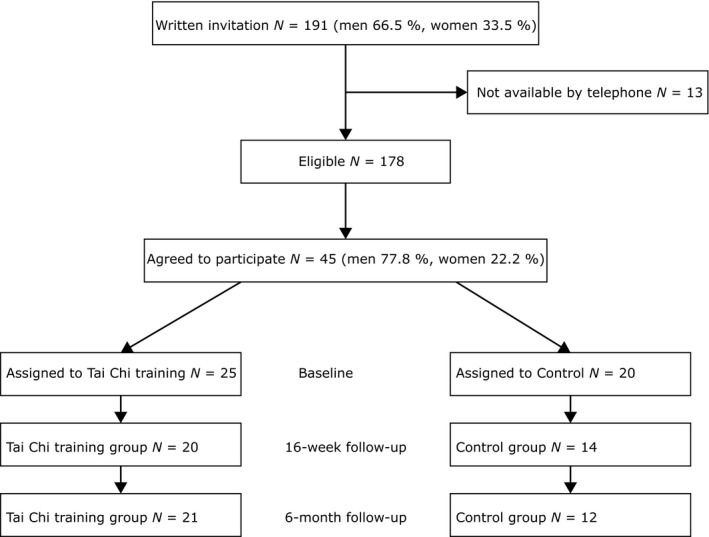
Flow chart of the recruitment and follow‐up procedure

At the time for baseline data collection, the participants were randomly assigned to either a control or training group. The intervention group underwent Tai Chi training twice a week for 16 weeks and the control group continued their normal living habits. Data were collected at baseline, at the end of the training period and at 6 months after the end of the training period, see Figure 1 (flowchart). Experienced physiotherapists and nurses tested the physical performance and asked for pharmacological changes and the participants filled in the questionnaires mentioned below. At the end of the training period, participants were asked if they were interested in participating in an individual interview concerning their experiences of the Tai Chi training.

## 4 INTERVENTION

The training programme was adjusted for people with heart failure by a doctor in traditional Chinese medicine, TCM. The training programme consisted of five movements from the Tai Chi Chuan simplified 24 forms, Yang style (cf. Li, Fisher, Harmer, & Shirai, 2003). The training groups were led by experienced Tai Chi leaders, one leader in each city. All were trained in this specific form by the investigator. The leaders also underwent a course in heart‐lung resuscitation before the intervention started. The classes lasted for 60 min, starting with a 10‐minute warm‐up. The whole programme could be performed sitting in a chair. At each session, a protocol was filled in by the leaders, covering attendance, performed activities and if any adverse episodes occurred.

Before the intervention started, a pilot group with seven (ten from start) participants, with the same inclusion criteria as the study group, tested the programme for 8 weeks. The researchers concluded that the training programme was suitable for this population and could be accomplished with some small revisions. The participants in the pilot group were excluded from invitation to the main study. The training for participants in the main study took place at a local training centre twice weekly during 16 weeks with an interruption of 3 weeks for Christmas holiday. From start there were six, seven and twelve participants, respectively, in the training groups.

## 5 OUTCOME MEASURES

*The Multidimensional Fatigue Inventory (MFI‐20)* (Smets, Garssen, Bonke, & de Haes, 1995) is a 20‐item self‐report instrument to measure fatigue covering five dimensions/subscales: General Fatigue, Physical Fatigue, Mental Fatigue, Reduced Motivation and Reduced Activity. Each subscale includes four statements with five possible answers, from agreement “yes that is true” to disagreement “no, that is not true”. The subscales are meant to be used separately with measures ranging from 4 to 20, with a higher score indicating more fatigue. The item statements refer to the last few days. MFI‐20 has been used in different samples of cancer patients and was found to have good internal consistency with Cronbach's alpha range 0.79–0.93 (Smets, Garssen, Cull, & De Haes, 1996; Smets et al., 1995). The instrument was translated into Swedish by Fürst and Åhsberg (2001) and the Swedish version has shown good internal consistency in studies among patients with cancer and congestive heart failure (Falk, Swedberg, Gaston‐Johansson, & Ekman, 2006; Fürst & Åhsberg, 2001; Hägglund, Boman, Olofsson, & Brulin, 2007).

*The Minnesota Living with Heart Failure Questionnaire (MLWHQ)* (Rector, Kubo, & Cohn, 1987) is a 21‐item self‐report instrument to measure the impact of living with heart failure on quality of life. The items cover statements about physical and psychological symptoms and functions and side effects of treatments. Possible answers are rated from 0 (no impact at all) – 5 (very high impact). All items are summed into a value ranging from 0 to 105, with a higher value indicating higher impact of heart failure on quality of life.

*The Short Physical Performance Battery – Swedish version (SPPB‐S)* (Guralnik et al., 1994) is a commonly used test measuring gait speed, standing balance and chair rise performance. Each part of the test is scored from 0 to 4 points with a summary score of 0–12; a higher value means better performance. The Swedish version was translated from English as a student project supervised by Professor Lillemor Lundin Olsson at Umeå University, Department of Community Medicine and Rehabilitation, with permission from Dr Guralnik and in collaboration with Dr Vestergaard, both from the National Institute on Aging, Laboratory of Epidemiology, Demography and Biometry, Bethesda, USA.

N‐terminal pro–Brain Natriuretic Peptide (NTproBNP) and BNP are the most important cardiac markers in patients with heart failure. It represents myocardial stretch due to volume or pressure overload. It has both high sensitivity and high specificity in the diagnostic procedure of heart failure. It is a very strong predictor for mortality and morbidity. Blood samples (plastic EDTA tubes) for analysis of NTproBNP were taken in fasting patients who had rested for 20 min. After 5 min, the samples were centrifuged for 10 min at 4°C and then stored frozen at –70°C. NTproBNP was analysed with Roche Elecsys proBNP immunoassay (Roche 2002).

## 6 STATISTICS

Mean values and standard deviations (*SD*) for baseline characteristics were calculated for continuous variables while natriuretic peptides were expressed as mean values and standard deviation (*SD*). Differences in continuous variables normally distributed data were evaluated by use of *t* tests. Chi‐square and Fisher's exact test was used for categorical data. The non‐parametric test, Mann‐Whitney U‐test was used for comparisons between groups for non‐normally distributed data and Wilcoxon signed rank test was used for related samples. Binary logistic regression was used for analyses within groups.

Power calculation was made based on earlier studies using the MLHFQ (Barrow et al., 2007; Yeh et al., 2004) and 66 participants were required to reach 80% power with an effect size of 33% (*p* < .05). Quantitative data were analysed statistically by using SPSS version 20.

## 7 INDIVIDUAL INTERVIEWS

An information letter was sent to 14 interested persons and finally ten of them (two women and eight men) participated in semi‐structured interviews. Participants from all three training groups were represented. The interviews were conducted by an experienced interviewer (RNT, who was not a member of the investigation team) and took place in the participants′ homes. The opening question was, “Please tell me about your experiences of participating in the Tai Chi training classes”. The following questions focused on health effects, positive or negative and how it had been to learn and perform the different movements. Finally, participants were asked what the training period meant to them and whether they intended to continue with Tai Chi after this period. The interviews lasted for 30–45 min, all were tape‐recorded and transcribed verbatim.

Qualitative data were analysed with content analysis inspired by Graneheim and Lundman (2004). The interview text was read through and meaning units related to the study's aim were identified. These units of text were then condensed and coded. Thereafter, codes were grouped into categories and an overall theme was formulated.

## 8 RESULTS

The mean age of the participants was 75 years, with a range of 71–85 years. A majority of the participants, 75%, had self‐rated scores above 12 on the MFI‐20 physical subscale (mean 13.4, *SD* 1.78), which might be interpreted as functional class (ll)‐lll on the NYHA classification scale. No participant reported previous experiences or knowledge about Tai Chi. Further background data is presented in Table 1 (baseline data).

**Table 1:** Baseline data

	Tai Chi training group *n*	Tai Chi training group Mean (*SD*)	Control Group *n*	Control group Mean (*SD*)	*p*‐value
Age Age range	25	75.6 71–85	20	75.5 71–83	.910
Men/women (*n*)	19/6		16/4		
Smoker (*n*)		2		0	
Earlier smoker (*n*)		14		12	
Chronic lung disease (*n*)		1		3	
Chronic joint pain (*n*)		3		2	
Stroke (*n*)		5		3	
BMI		25.8		28.4	.055
6‐min walk‐test, m		393		358	.258
Balance‐test total SPPB		8.7		8.5	.735
balance		3.7		3.7	.538
walk		3.6		3.3	.309
upraising		1.4		1.6	.758
General fatigue		10.7		11.6	.404
Physical fatigue		13.5		13.3	.553
Mental fatigue		10.3		11.3	.034^a^
Reduced activity		12.3		12.8	.776
Reduced motivation		12.9		12.4	.540
MLHFQ total sum (max 105)	18	39.4 (22.6)	14	45.2 (24.3)	.377

a *p* < .05.

The attendance at the Tai Chi training sessions was as follows: 18 of 25 participants completed 75% or more of the sessions and seven participants completed less than 75%. There were no statistically significant differences between those above and those below 75% attendance at baseline for any variable. No adverse events were reported during the training sessions.

## 9 EFFECTS OF THE TAI CHI GROUP TRAINING ON FATIGUE, QUALITY OF LIFE, PHYSICAL PERFORMANCE AND NATRIURETIC PEPTIDES

### 9.1 Comparisons between groups

At baseline, participants in the control group rated a higher degree of mental fatigue compared with the training group (mean 11.3 vs. 10.3, *p* = .034) and had higher BMI than participants in the training group (mean 28 vs. 26, *p* = .055). At the end of the training period, 16 weeks after baseline, data were available from 21 participants in the training group and 14 participants in the control group. A borderline significant difference between the groups was found in one of the MFI‐20 subscales where participants in the training group rated more reduced activity (13.1 vs. 11.8, *p* = .056).

At the 6‐month follow‐up after the training period, participants in the control group rated more mental fatigue than those in the training group (11.8 vs. 10.5, *p* = .048). At this time, data were available from 21 participants in the training group and 14 participants in the control group.

### 9.2 Comparisons within groups

Participants in the training group reported more general fatigue at the 16‐week follow‐up (mean 11.4 *p* = .036) and at 6 months (mean 11.3 *p* = .042) compared with baseline (mean 10.7). No other item showed any statistically significant differences over time.

For the participants in the control group, there was an improvement in rising from a chair from baseline to the 16‐week follow‐up (mean 1.6 vs. 2.6 *p* = .033) and for the Short Physical Performance Battery (SPPB) total sum from baseline to 6‐month follow‐up (mean 8.5 vs. 9.9 *p* = .046). No other item showed statistically significant differences over time.

Natriuretic peptides: The mean values of NTproBNP were 4479 ± 7309 ng/L and 3379 ± 7332 ng/L (*p* = 0,90) in the Tai Chi group and the control group, respectively. At the 16‐week follow‐up, the mean values were 3279 ± 3448 ng/L and 2736 ± 2594 ng/L (*p* = .81). A within group analysis revealed that NTproBNP decreased in both groups for the Tai Chi group from 3656 ± 7446 ng/L to 3280 ± 3448 ng/L (*p* = .058) and from 3044 ± 2252 to 2736 ± 2595 (*p* = .583) in the control group.

## 10 PATIENTS’ EXPERIENCES OF PARTICIPATING IN TAI CHI GROUP TRAINING

Results from qualitative interviews about participating in Tai Chi group training are presented in one comprehensive theme with four underlying categories.

### 10.1 Theme: Finding a new, feasible and meaningful activity

Participants stated that participating in the Tai Chi training class was a positive experience. Tai Chi was seen as possible to learn and perform with a group of people who have similar health status and which was led by a skilled leader. Reported health effects were improvements in balance and feeling better from the breathing exercises. Informants also expressed a wish to continue in a training group after this period ended.

### 10.2 Category 1: To learn and perform the Tai Chi movements

This was an unknown form of training and some were initially doubtful about their bodily capacity to perform the movements and if this kind of training could be helpful:

I've had so much else… I've had cancer of the throat, cancer of the abdomen… vertebral compressions and I thought, “No… I wonder if my body will manage this” but it did… (3)

Participants appreciated that the Tai Chi training was new for all the participants. After some time, it felt nice and stimulating, not too strenuous and possible to perform despite heart failure and other comorbidities. The single movements were not considered to be hard to learn and perform, but to coordinate different movements while thinking about breathing patterns was more difficult. It was not easy to remember the different movement series, but after repeatedly training in smaller pieces the performance became better and better. To see the leader and other participants doing the movements helped and continuity in training was important:

I said from the beginning that it's toughest for the head… less tough for the body (6)

Some informants had difficulties with balancing, especially with the slow pace of Tai Chi. One woman placed herself near a window so she could hold the windowsill when needed. Calm Chinese music was added during the final sessions and some thought it was helpful to get the right pace, but one man was disturbed by the music. Tai Chi was seen as a suitable form of physical activity, especially in winter when it can be problematic to move outdoors.

### 10.3 Category 2: The importance of the leader and the group

The leader (although this was three different people) was described as good, competent, nice, patient and humorous. The leader had a very important role in both teaching Tai Chi as well as creating a good atmosphere. Being part of a group when learning and performing Tai Chi was considered vitally important:

we were beginners, all of us… we… we learned because… but I think it is easier if you're a group…(4)

Informants described a positive atmosphere in the group, such as joking and laughter. It was meaningful to meet others with similar health problems. To get to the group sessions was a goal in its own, a reason to get out and maybe have a walk to and from the classes. Some informants felt that the social interaction was the best aspect of the Tai Chi training period. However, difficulties with getting to the classes due to travelling a long distance, transport problems or weather was reported as hindering participation in group sessions.

### 10.4 Category 3: Perceptions of health in relation to Tai Chi training

Nobody spoke about physical discomfort or risk of injuries, other than being stiff. Some did not experience any differences in health, but others said they felt better. Improvements in balance were most frequently mentioned and feeling better from the breathing exercises was reported. Other effects were being calmer, feeling a bit tired or feeling fit after a training session. Informants also observed health improvements among other participants:

Well, what I knew that directly affected it… is this thing with the balance – it's… like, you started to walk and shuffle your feet… [laugh]… like that – you've become an old man – but I think you got rid of that… through the balance training (5)

### 10.5 Category 4: Tai Chi training at home and other physical activities

Informants spoke of trying to perform Tai Chi at home, at least some movements as it was hard to remember the whole session. One woman said that it would have been good to have tape‐recorded instructions to use at home and many wanted the group training to continue after the end of the study period. The movements that were practiced at home focused on breathing and balance, but most informants said that they rarely or never practiced Tai Chi after the Tai Chi training period had ended. One man pointed out that he wanted to be alone while practicing Tai Chi, with nobody watching him. Some informants used to be physically active by walking, playing boule or using rubber bands for muscle strengthening. One man said he preferred the usual physiotherapy, but also kept on practicing some of the Tai Chi movements:

but then there were a few movements… it was… the breathing exercises, I still do them every day, several times a day… and then there was a balance number she would have us do… [laugh]… that… I practice too (7)

## 11 DISCUSSION

The findings from the quantitative part of this study did not support our hypothesis as there was no statistically significant differences in fatigue, quality of life, physical performance or NTproBNP between the two studied groups. In contrast to what was expected, the training group tended to report higher levels of reduced activity after the 16 weeks of training, compared with the control group. Also analyses within the two groups over time are in line with the differences between groups. However, due to the small sample size these findings may have occurred at random. The lack of improvement in above‐mentioned variables in this study differs from findings from the meta‐analysis by Gu et al. (2017) covering studies among patients with CHF. One possible reason for these differences could be that participants in this study had a higher mean age. Unfortunately, there is insufficient information to compare other important factors such as levels of NYHA classification or comorbidity.

The main finding from the qualitative part of this study is positive experiences from the Tai Chi training. The participants were able to learn and perform the movements. Many participants emphasized their improved balance, which is in line with earlier research. In a qualitative study, CHF patients reported among other things improvement in self‐efficacy, specifically in performing exercise and also feelings of empowerment and control (Yeh, Chan, Wayne, & Conboy, 2016). The social engagement of the group and the influence of the leader was reported to be important. Despite hindrances due to cold winter weather with heavy snowfall, the attendance rate indicated that participants were anxious to come to the Tai Chi class. Another factor that could contribute to their positive experiences is the low pace and low intensity of the movements of Tai Chi, which can be different from other activities. Although the lower intensity Yeh et al. (2013) found benefits of Tai Chi similar to other aerobic exercises in a small group of patients with CHF.

The meaning of leader‐led supervision and group fellows for adherence to physical exercises has been reported earlier (Tierney et al., 2011). However, many patients with HF can have difficulties getting to group sessions due to health problems or other practical impediments. One way to overcome such problems is to participate through some kind of telecommunication, which has been successfully evaluated among older people (Silveira, van het Reve, Daniel, & Casati, 2013; Wu, Keyes, Callas, Ren, & Bookchin, 2010). The use of IT‐based systems in this area seems to have great potential for the future.

Concerning compliance to advice for self‐care the influence of patients′ knowledge and beliefs need to be accounted for, not the least for physical activity where compliance is reported to be low (van der Wal et al., 2006). Rogers, Keller, and Larkey (2010) reported from a review of studies among older people (mean age 67 years, mostly women) that the reasons for beginning and continuing Tai Chi were perceived health benefits and socialization. In this study none of the participants had earlier experiences of Tai Chi and they were not asked about their expectations and thoughts about Tai Chi at baseline, which could have been of interest. To minimize influences on their expectation of the intervention the leaders did not teach about the philosophy and history of Tai Chi, or about expected health benefits from the exercise. The qualitative interviews show there was an interest to continue with Tai Chi. However, one must consider the possibility that it was those who had the most positive experiences of Tai Chi who accepted the invitation to be interviewed.

### 11.1 Methodological considerations

Of the 191 patients who fulfilled the inclusion criteria only 45 agreed to participate in the study, which markedly reduced the power of this study. Patients were not asked for the reasons why they did not want to participate or dropped out of the study, yet some patients and/or relatives explained their reasons. Common reasons given for non‐participation were health problems, difficulties with getting to the classes, cold weather, language difficulties and other activities such as travelling or family matters. Of those invited to the study up to 67% were men and in the group of participants the proportion of men was even higher, 78%. We could have expected a higher proportion of women in this population as CHF is as or more common among women in this age group (Olofsson et al., 2007) than in men.

Despite weak statistical power, the random distribution of participants into the two study groups should be considered a strength. Randomization probably minimizes the risk of bias caused by participants’ attitudes since this is reported to be an important factor for attendance to exercise classes among older adults (Hawley‐Hague et al., 2014). Besides the weak power due to the small sample size, another important explanation of the study outcome was differences in drop‐out rates and lost to follow up. In the training group 20 and 21, respectively, of 25 participants completed the two follow‐up tests, while in the control group only 14 and 12, respectively, out of 20 participants completed these tests. However, the relatively high attendance rate in the Tai Chi group could be considered as a strength. The sample size is too small for statistical adjustments of differences at baseline between those who completed the follow‐up tests and those who dropped out. Therefore, it is impossible to know the magnitude of how the drop‐out rate influenced the results. In an evidence map of health outcomes from Tai Chi studies in different settings, Solloway et al. (2016) find promising results, but due to methodological limitations and insufficient number of studies, there is still a need of future powered clinical trials. The mixed methods design gave complementary knowledge concerning Tai Chi group training for older patients with CHF. Results from both methods show that Tai Chi did not change health perception significantly, but qualitative data showed important benefits in balance that was not seen in statistical analysis. The findings of increased fatigue and reduction in activity in the Tai Chi training group are not expressed in the interviews. However, it might be possible that those who agreed to participate in individual interviews were more alert than those who refrained from being interviewed. The findings of this study may illustrate the value of combining quantitative and qualitative research methods. The choice of outcome variables can be questioned. There may be other factors of importance for older patients with CHF to be considered for evaluating Tai Chi group training. The comprehensive theme from the individual interviews is that Tai Chi group training is experienced as feasible and meaningful for patients with CHF. Further investigations using feasibility and meaningfulness as outcome variables seem to be of interest.

In line with the concept of person‐centred care, it is important to make health care plans together with the patient and their relatives if possible, which are based on their values and preferences (Ekman et al., 2011). Tai Chi offers an alternative of exercise at low intensity and a good opportunity to combine the positive experience of group activity with the possibility of home‐based practice, as well as through the use of IT‐technology.

## 12 CONCLUSION

No statistically significant beneficial effects on quality of life and physical performance was seen after 16 weeks Tai Chi training among patients with CHF aged over 70 years, compared with a control group. These findings may be due to lack of effect or that our study was underpowered to show clinically relevant effects. Qualitative data showed that Tai Chi training was experienced to be feasible and meaningful. It was reassuring that there were no reported adverse episodes. The mixed method approach contributed to a more fully insight in participants experience of Tai Chi training. For the purpose to increase adherence to physical activity in this frail population, future studies should focus on apprehension of motivating factors as feasibility and meaning.

## 13 RELEVANCE TO CLINICAL PRACTICE

Physical exercise is recommended for patients with CHF. However, common impediments like sedentary lifestyle, changes due to old age, comorbidity or advanced CHF can make it difficult to follow this recommendation. With a view to performing person‐centred care, it is important to find activities suitable to the individual capacity and interests. Tai Chi training in a programme adjusted for patients with heart failure may be an alternative of physical exercise for patients aged over 70 years. Tai Chi training and can easily be performed in group sessions or at home.

## ETHICAL APPROVAL

The study was approved by the Regional Ethical Review Board, Umeå, reference number 09‐116M and 2010‐279‐32M, and registered at Clinical Trials.gov NCT01294111.

## AUTHOR CONTRIBUTION

Lena Hägglund was responsible for the design of the study. She organized the intervention and data collection, contributed to data analysis and the drafting of the manuscript. Kurt Boman contributed to the study design, the interpretation of data and the critical revision of the manuscript. Margareta Brännström contributed to the study design, the interpretation of data and the critical revision of the manuscript.
